# Liposome-Encapsulated ISMN: A Novel Nitric Oxide-Based Therapeutic Agent against *Staphylococcus aureus* Biofilms

**DOI:** 10.1371/journal.pone.0092117

**Published:** 2014-03-21

**Authors:** Camille Jardeleza, Shasha Rao, Benjamin Thierry, Pratik Gajjar, Sarah Vreugde, Clive A. Prestidge, Peter-John Wormald

**Affiliations:** 1 Department of Surgery- Otorhinolaryngology Head and Neck Surgery, The Queen Elizabeth Hospital, and the University of Adelaide, Adelaide, South Australia; 2 The Ian Wark Institute, University of South Australia, Mawson Lakes, South Australia; Xi'an Jiaotong University School of Medicine, China

## Abstract

**Background:**

*Staphylococcus aureus* in its biofilm form has been associated with recalcitrant chronic rhinosinusitis with significant resistance to conventional therapies. This study aims to determine if liposomal-encapsulation of a precursor of the naturally occurring antimicrobial nitric oxide (NO) enhances its desired anti-biofilm effects against *S. aureus*, in the hope that improving its efficacy can provide an effective topical agent for future clinical use.

**Methodology:**

*S. aureus* ATCC 25923 biofilms were grown *in-vitro* using the Minimum Biofilm Eradication Concentration (MBEC) device and exposed to 3 and 60 mg/mL of the NO donor isosorbide mononitrate (ISMN) encapsulated into different anionic liposomal formulations based on particle size (unilamellar ULV, multilamellar MLV) and lipid content (5 and 25 mM) at 24 h and 5 min exposure times. Biofilms were viewed using *Live-Dead* Baclight stain and confocal scanning laser microscopy and quantified using the software COMSTAT2.

**Results:**

At 3 and 60 mg/mL, ISMN-ULV liposomes had comparable and significant anti-biofilm effects compared to untreated control at 24 h exposure (p = 0.012 and 0.02 respectively). ULV blanks also had significant anti-biofilm effects at both 24 h and 5 min exposure (p = 0.02 and 0.047 respectively). At 5 min exposure, 60 mg/mL ISMN-MLV liposomes appeared to have greater anti-biofilm effects compared to pure ISMN or ULV particles. Increasing liposomal lipid content improved the anti-biofilm efficacy of both MLV and ULVs at 5 min exposure.

**Conclusion:**

Liposome-encapsulated “nitric oxide” is highly effective in eradicating *S. aureus* biofilms *in-vitro*, giving great promise for use in the clinical setting to treat this burdensome infection. Further studies however are needed to assess its safety and efficacy *in-vivo* before clinical translation is attempted.

## Introduction

The battle against bacterial infections persists despite discoveries of new and broader spectrum antibiotics. Over use of antibiotics promotes the emergence of drug-resistant strains which makes successful treatment even more challenging. Thus the global quest for better and more efficacious antimicrobial agents continues.

*Staphylococcus aureus* (*S. aureus*) remains one of the most commonly known opportunistic pathogens colonizing up to 2 billion people worldwide. Ranging from chronic skin infections[1] and allergic dermatitis,[2] to osteomyelitis[3] and sepsis,[4] *S. aureus* has great versatility in contributing to diseased states in both healthy and immunocompromised individuals[5]. Its existence in biofilm form has been well documented,[6], [7] partially explaining the difficulty in eradication and its ability to cause a repeated cycle of infection. Encased in an extra-polymeric substance matrix which enhances microbial survival and impairs antimicrobial penetration,[8] bacterial biofilms require up to 1000x greater antibiotic dose for effective treatment compared to their planktonic counterparts.[9] This contributes to treatment failure and emphasizes the need for the development of alternative antimicrobial treatment strategies.

In particular, the isolation of *S. aureus* biofilms in the sinuses of patients with chronic rhinosinusitis (CRS) has been linked to poorer clinical outcomes and disease prognosis[10]–[12], further defining the importance of this microorganism in disease severity. Although antibiotics initially improve patients’ signs and symptoms, recurrence after treatment cessation is often the case, demonstrating the limitations of the use of antimicrobial compounds.

In the sinuses of healthy individuals, the endogenous gas nitric oxide (NO) has been documented to be highly concentrated, sometimes reaching maximum allowable air pollutant levels,[13] while the levels found in CRS patients are significantly lower.[14]–[16] NO has demonstrated antibacterial and antiviral properties[17], plays a role in innate immunity and mucociliary clearance[18] and is thought to provide a significant contribution to maintaining the balance of normal flora in non-diseased sinuses. Recent evidence has shown NO to also have anti-biofilm effects against a wide range of organisms[19], including *S. aureus*.[20] Its natural occurrence in the body in conjunction with its antimicrobial properties gives it great potential for topical application against *S. aureus* biofilm-associated infections, both within and outside the confines of the paranasal sinuses.

Whereas high NO levels have been shown to have anti-biofilm effects on established *S. aureus* biofilms, we have demonstrated that low NO levels increase the quantity of a *S. aureus* biofilm biomass.[21] The dualistic effect of NO on *S. aureus* biofilms indicates that the desired anti-biofilm effects are critically dependent on the NO concentration and exposure time.[21] Therefore there is a need to improve NO’s formulation prior to clinical application as a topical therapy. Isosorbide mononitrate (ISMN), a clinically approved NO-donor, has a well-established safety profile in a range of applications.[22], [23] Conversely, a number of liposome-based drugs are already clinically approved thereby demonstrating safety for clinical use.[24], [25] Liposomes typically increase drug specificity, lessen the risk of adverse drug reactions, decrease the required dose and prolong drug-release, all ideal properties of a suitable topical anti-biofilm agent. In addition, liposomes have been found to be effective in delivering antibiotics and other therapeutics to various biofilms.[26], [27] Importantly, the inherent bactericidal and anti-biofilm effects of cationic and anionic liposomes are well demonstrated.[28]–[30] This motivated the design of a novel ISMN liposomal formulation towards the development of a novel topical treatment against *S. aureus* biofilms with potential clinical applications in *S. aureus* biofilm-associated diseases such as CRS. We specifically aimed to determine the synergistic anti-biofilm effects obtained with unilamellar and multilamellar anionic liposomal (ULV and MLV respectively) formulations of ISMN. To this end, anionic ULV and MLV liposomes were prepared and their efficacy tested *in vitro*.

## Methodology

### Nitric oxide donor

99% pure grade ISMN (Bosche Sci, NB, New Jersey) was used as the NO donor. ISMN was chosen for liposomal encapsulation due to its well-documented side effect profile and its established safety for human use in the field of cardiology.[22], [23] Characterization of the anti-biofilm and anti-planktonic effects of the free drug form was first performed. Since the maximum aqueous solubility of ISMN was at 60 mg/mL, serial dilutions down to concentrations of 3.75 mg/mL were tested for anti-biofilm effects. CSF broth was used as the diluent to ensure that it was not the absence of bacterial culture media that was causing the desired effect. Anti-planktonic properties were obtained by reading the optical density (OD) at 540 nm at the bottom of each well of the challenge plate.

### Culture and formation of *S. aureus* biofilms

A biofilm forming reference strain *S. aureus* American Type Culture Collection (ATCC) 25923 was used to test against the different liposomal-NO formulations. *S. aureus* culture and biofilm formation was performed as previously described.[21] Briefly, a single loop of *S. aureus* glycerol stock was defrosted at 37°C in 2 mL of cerebrospinal fluid (CSF) broth (Oxoid, Australia) for 18–24 hours under agitation. 1 loop of culture was then plated onto a blood agar plate (Oxoid, Australia) and incubated for 18–24 hours at 37°C, following which 1–2 bacterial colonies were immersed in 0.45% saline to create a 1 McFarland unit (MFU) solution (3×10^8^ colony forming units/mL). This solution was then diluted to 1∶15 in CSF broth, 150 μL of which was pipetted into each well of a 96-well plate of the Minimum Biofilm Eradication Concentration (MBEC) biofilm-forming device (Edmonton, Canada).

The MBEC device was used as per manufacturer’s instructions. The pegs suspended on the lid were immersed into the 96-well plate containing the bacterial solution and incubated for 44 hours at 35°C on a gyrorotary shaker (Ratek, Vic, Aus) at 70 rpm, allowing the biofilms to form on each peg’s surface.

### Liposomal preparation

Anionic liposomes were prepared containing egg lecithin:dipalmitoylglycero-phosphoglycerol (DPPG) at 4∶1 mol ratio. The required amount of lipids was weighed into a 25 ml round bottom flask and dissolved in 5 ml of chloroform. The chloroform was slowly removed under reduced pressure using a Buchi rotary evaporator (Buchi, Germany) to deposit a thin film of dry lipid on the inner wall of the flask. The dry lipid film was hydrated with 5 ml of blank phosphate-buffered saline (PBS) solution or PBS solution containing either 3 mg/ml or 60 mg/ml of ISMN for at least 1.5 hours at a temperature of 5°C above the phase transition temperature of the main lipid to obtain the MLV vesicles. ULV were produced from MLVs by extrusion through 800 nm, 400 nm and 200 nm pore size polycarbonate membranes in a Lipex 10 ml Thermobarrel Extruder (Burnaby, BC Canada). In the anti-biofilm activity studies, freshly prepared ULV and MLV ISMN liposomal formulations were used without purification. The latter approach was selected to increase the translational potential of the proposed ISMN liposomal formulations.

### Liposome Characterization

#### Particle Size Analysis

The particle size of the blank liposomes and ISMN-loaded liposomes were characterised using a dynamic light scattering (DLS) technique which has a size detection range of 0.6 nm to 6 μm (Malvern Zetasizer Nano ZS, UK). Liposomes were diluted 100-fold with 10 mM sodium chloride (NaCl) aqueous solution prior to measurement at 25°C. Water (refractive index  =  1.33) was used as the dispersant in the DLS analysis. A typical liposome refractive index of 1.45 was used.[31] Size distribution results are expressed as the z-average diameter (*i.e.* the intensity-weighted mean hydrodynamic diameter) together with the polydispersity index (PDI) indicating the width of the size distribution.

#### Determination of Zeta-potential

Liposomes were diluted 100-fold with 10 mM NaCl aqueous solution prior to the measurement of zeta potentials. Zeta potentials were determined by a using phase analysis light scattering (PALS) technique (Malvern Zetasizer Nano ZS, UK) at 25°C, with the detection limit of 5 nm to 10 nm particles. The mean zeta potential was computed based on the electrophoretic mobility (*i.e.* the ratio of the velocity of particles to the field strength) by applying the Smoluchowski or Huckel theories.

#### Determination of drug encapsulation efficacy

Liposomes were ultra-centrifuged at 30,000 rpm at 4°C for one hour. The supernatant was taken and diluted with mobile phase and analysed by high performance liquid chromatography (HPLC) to determine the amount of free drug (***C_free_***). The pellet phase was rehydrated in 1 mM PBS. 0.2% Triton X-100 was added to break the phospholipid structure and free the entrapped drug. The mixture was sonicated (30 min) and centrifuged at 20,000 rpm for 30min. The supernatant was taken, diluted and analysed by HPLC to determine the amount of encapsulated drug (***C_encapsulated_***). The encapsulation efficiency (***En%***) was calculated using the following equation: 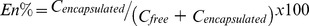



### HPLC Analysis of ISMN

An HPLC method employing UV detection was used for quantification of ISMN-containing samples (Shimadzu, Japan). Chromatographic separation was performed on a LiChrospher RP C_18_ column (5 μm, 4.6 mm ID×150 mm, Grace Davison Discovery Science, Rowville, VIC) at a detection wavelength of 196 nm. The mobile phase was a mixture of methanol and water (20:80 v/v), eluded at a flow rate of 1.5 ml/min. The sample was injected at a volume of 50 μl at 40°C. The average retention time was 3.4 min; detection limit of the method was 15 ng/ml. The linearity range of the method used was 0.1–10 μg/ml with an R^2^ (correlation coefficient) value of 0.998. Within-day precision was <3% and between-day precision was <4%.

### Exposure of *S. aureus* biofilms to the Liposome-encapsulated ISMN

All liposomal experiments were repeated twice. The *S. aureus* biofilm-coated pegs prepared using the MBEC device were washed for 1 minute in 1x PBS to remove planktonic bacteria and exposed to a challenge plate containing 180 μL of the liposomal test agent added with 20 μL of 0.4 mM L-Arginine (Musashi, Vic Aus) to mimic a bacterial culture state[32] for 24 hours at 37°C. Based on these results, the liposomal formulations with the best anti-biofilm effects were selected and tested at a shorter exposure time of 5 minutes, chosen to better simulate the rapid exposure time of topical douching into the sinuses. After 5 minutes of exposure to the challenge plate, the pegs were re-immersed into a new 96-well plate containing 180 μL of CSF broth with 20 μL of 0.4 mM L-Arginine and incubated for 24h.

### Biofilm Imaging and Quantification

After treatment exposure, the pegs were washed twice in 0.9% NaCl for 1 minute and 10 seconds respectively to remove planktonic bacteria as per manufacturer’s instructions. These were then fixed in 5% glutaraldehyde (Sigma Aldrich, St Louis MO) for 45 minutes, followed by a repeat wash in 0.9% NaCl for 10 seconds to remove excess fixative. The pegs were then individually placed in 1 mL sterile milliQ water (Millipore, Billerica, MA) containing 1.5 μL each of the *LiveDead* Baclight stains (Invitrogen Molecular Probes, Vic Aus) Syto 9 and propidium iodide and incubated on a rotating mixer at 10 rpm (Pelco, CA USA) in the dark at room temperature for 15 minutes. The pegs were again rinsed in 0.9% NaCl for 10 seconds and individually mounted on cover slips for viewing under the Leica TCS SP5 confocal scanning laser microscope (Leica Microsystems, Wetzlar, Germany).

2 representative z-stacks each containing 120 +/– 5 serial images set at 0.7 μm distance between 2 images were obtained per peg. Biofilm quantification of each z-stack was then calculated using the COMSTAT 2 software.[33]

### Statistical Analysis

Graphpad Prism 5.0 (San Diego Ca) was used to calculate one-way analysis of variance (ANOVA) when comparing biofilm biomass of more than 2 treatment groups with Bonferroni multiple comparisons (95% confidence interval) as post hoc test using the R statistical software (R Foundation for statistical computing, Vienna, Austria). Unpaired t-test was used to compare 2 groups for the anti-biofilm effects of the pure drug ISMN. A p value of <0.05 was considered statistically significant.

## Results

### Characterization of Liposomes

Anionic liposomes were prepared from Egg lecithin and DPPG (4∶1 molar ratio) using the standard thin film hydration method. ULV liposomes were prepared from MLV’s using standard extrusion. The physicochemical properties of the samples, namely hydrodynamic diameter and zeta-potential, were obtained using dynamic light scattering and are presented in **Table 1**. The average hydrodynamic diameter of the as prepared MLV was 692 nm (PdI =  0.7). After membrane extrusion, ULV liposomes were obtained with an average hydrodynamic diameter of 351 nm (PdI =  0.6). The incorporation of ISMN during the hydration step had little effect on the size of the liposomes, although a small reduction was observed for the ISMN loaded MLV liposomes (536 nm, PdI =  0.8) and a small increase was observed for the ISMN loaded ULV liposomes (384 nm, PDI =  0.2). The zeta potential was measured in 10^−2^ M NaCl and as expected all liposomal preparations displayed negative potentials. The zeta potential values have been shown to be sufficient with excellent colloidal stability.

**Table 1:** Particle size and zeta-potential of the prepared anionic liposomes.

Liposome	Lipid Composition	Hydrodynamic diameter (nm)		Zeta Potential (mV)	
		MLV	ULV	MLV	ULV
Blank	Egg lecithin : DPPG 4 : 1	692	351	–27.7	–38.5
ISMN		536	384	–32.9	–19.6

ISMN: isosorbide mononitrate; MLV: multilamellar; ULV: unilamellar

Next the ISMN encapsulation efficiency was determined using HPLC at two lipid concentrations, 5 mM and 25 mM. As expected, the encapsulation efficiency was higher at higher lipid concentrations for both ULV (1.3% at 5 mM vs. 6.3% at 25 mM) and MLV (1.5% at 5 mM vs. 10.7% at 25 mM) liposomes (**Table 2**), while the increase in the lipid concentration had no significant impact on the particle size.

**Table 2:** Drug encapsulation efficiency of anionic liposomes loaded with 60/mL of isosorbide mononitrate.

Liposome	En%	
	MLV	ULV
Ani-ISMN-5 mM	1.47±0.32	1.30±0.19
Ani-ISMN-25 mM	10.73±1.51	6.34±0.19

MLV: multilamellar; ULV: unilamellar; En%: percentage of drug encapsulated.

### Anti-biofilm and anti-planktonic effects of ISMN (free-drug)

**Figure 1:**
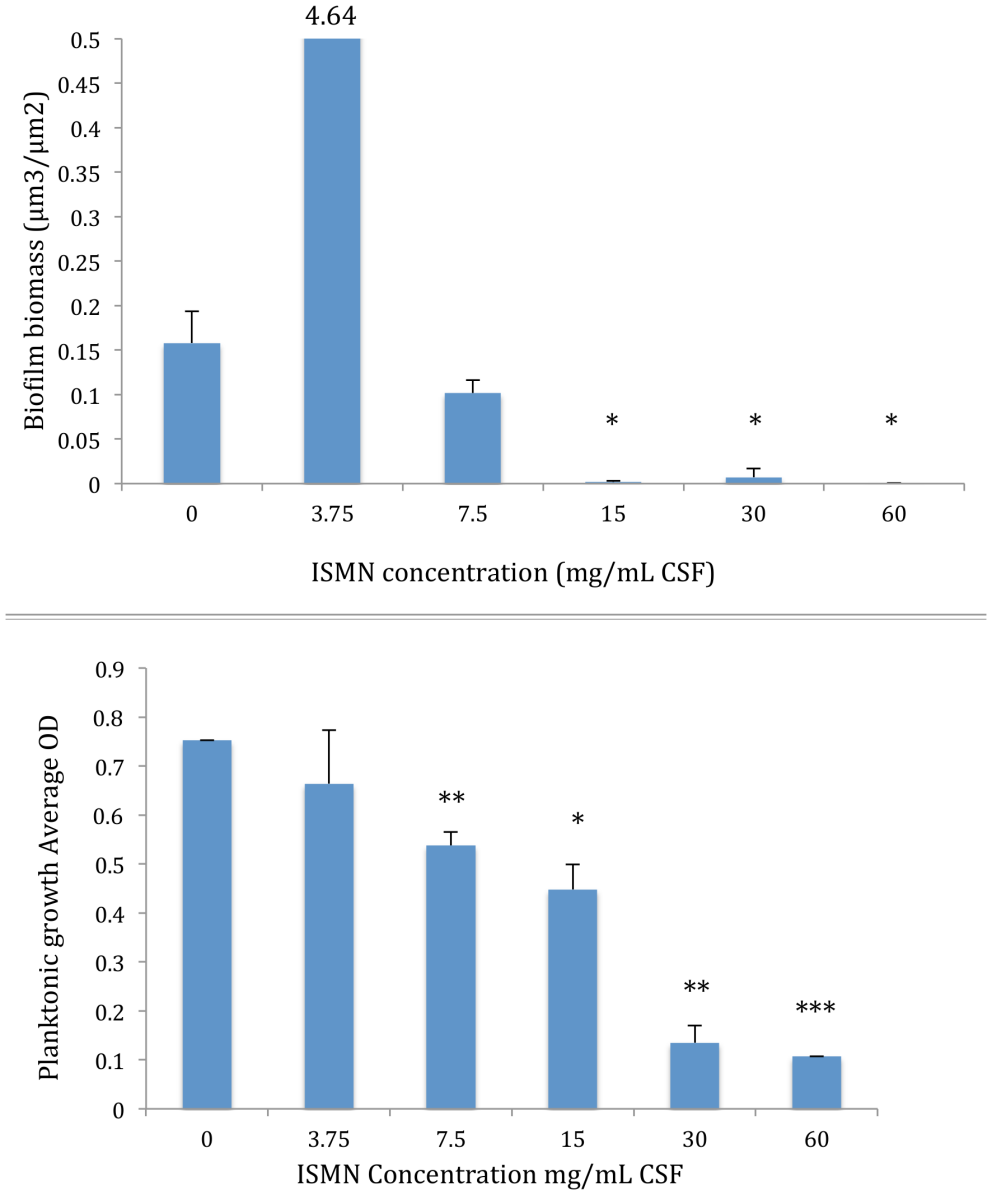
Dose-response of pure ISMN on *S. aureus* biofilm biomass (A) and planktonic cell growth (B). 4.64 is the biofilm biomass at 3.75 mg/mL ISMN concentration. ISMN: Isosorbide mononitrate. Data represents Mean +/– SD of a duplicate experiment. *, P<0.05; **, P<0.01, ***, P<0.001 compared to untreated control; Two-tailed *t* test.

There was variability of biofilm growth of the untreated control pegs across all experimental runs. These findings are similar to our previous in-vitro biofilm studies,[21] and is attributed in part to the stochastic process of biofilm development despite maintaining constant growth conditions.[33] One-way ANOVA failed to show statistical significance when comparing all treatment groups. A global overview however, shows that biofilm growth at the lowest ISMN concentration of 3.75 mg/mL was increased, compared to the untreated control peg. (**Figure 1**) The unpaired t-test was then used to determine if statistical significance was present when comparing 2 groups. There was still no statistical significance between the 3.75 mg/mL ISMN vs. untreated control (unpaired t-test, p =  0.21). However, subsequent and increasing concentrations of ISMN showed anti-biofilm effects, resulting in an almost complete eradication at 60 mg/mL ISMN dose with statistical significance (unpaired t-test vs. control, p =  0.024). This paradoxical pattern of enhanced biofilm growth at low NO concentrations and anti-biofilm effects at higher concentrations is consistent with findings from our previous studies using the NO donor DetaNONOate.[21] The lowest ISMN concentration with statistically significant anti-biofilm effects compared to the untreated control was at 15 mg/mL (unpaired t-test vs. control, p =  0.025).

The anti-planktonic effects were also tested in the free-drug form to completely characterize ISMN’s antibacterial effects in order to obtain the best possible dose for liposomal encapsulation. Planktonic growth is shown to be inversely proportional to ISMN concentrations with the greatest anti-planktonic effect at 60 mg/mL (One way ANOVA, p =  <0.0001). **Figure 1** summarizes these findings.

### Anti-biofilm efficacy of ISMN liposomal formulations at 24-hour exposure

To establish the anti-biofilm potential of the ISMN-Liposomal formulations, anionic ULV liposomes prepared with different ISMN doses (3 mg/mL and 60 mg/mL) were tested using initially a contact time of 24 hours. Their anti-biofilm activity was compared to untreated control pegs (*S. aureus* biofilms grown for 44 hours on pegs immersed in pure CSF broth), corresponding ULV blanks, and liposome-free treated control of pure drug 60 mg/mL ISMN.

**Figure 2:**
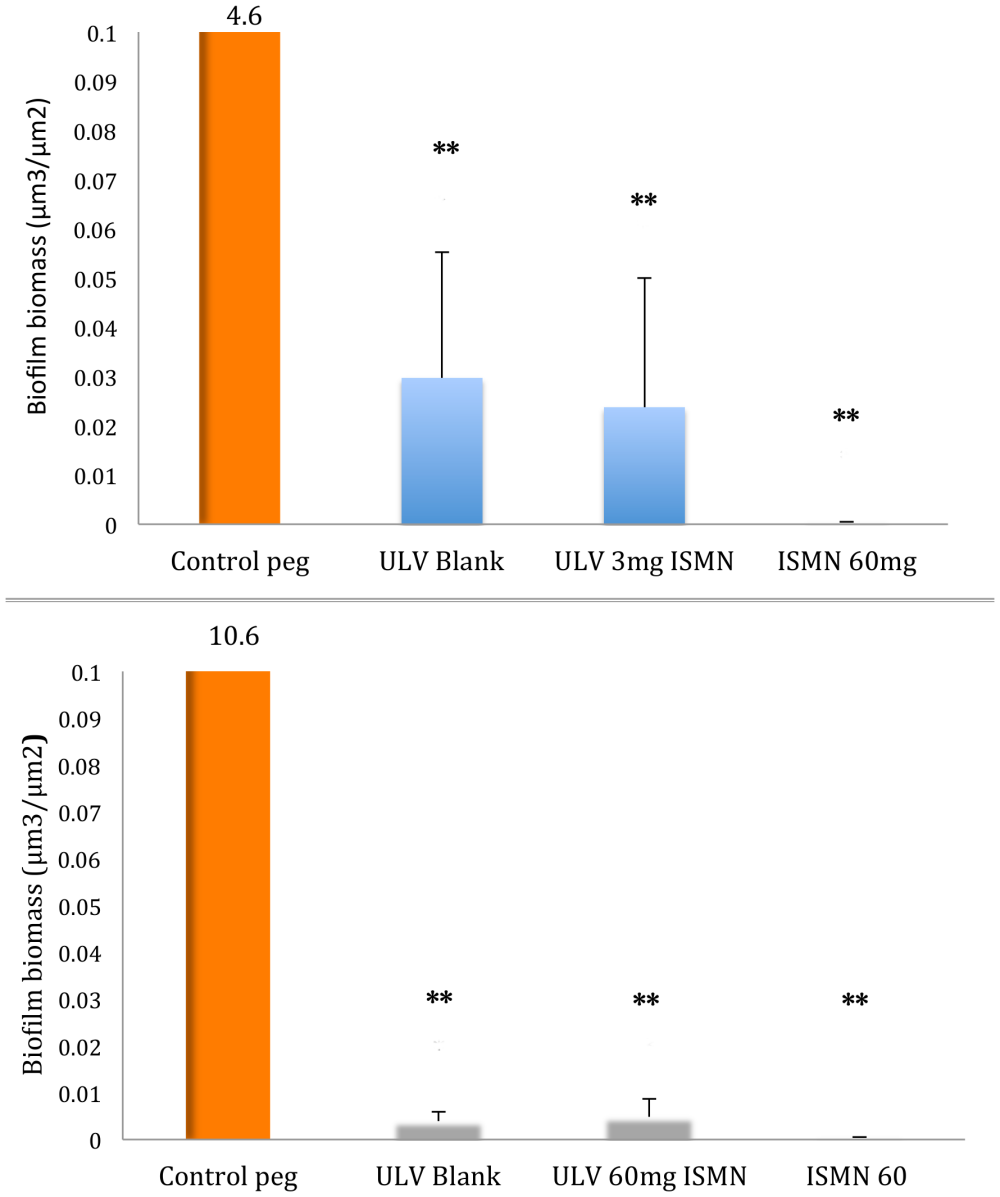
Effects of ISMN, ULV Blank and ISMN-ULV liposomes on *S. aureus* biofilm biomass after 24 hours. The numbers above the bar represents the biofilm biomass of the untreated controls. ULV: Unilamellar liposome; ISMN: Isosorbide mononitrate. Data represents Mean +/– SD of a duplicate experiment. A replicate experiment showed the same trend. *, P<0.05; **, P<0.01, ***, P<0.001 vs untreated control; Bonferroni’s multiple comparisons test.

The results are summarized in **Figure 2**. One-way ANOVA using Bonferroni comparisons test showed that there was a significant decrease in biofilm biomass in pegs treated with both the 3 mg/mL ISMN and 60 mg/mL ISMN ULV liposomal formulations when compared to their corresponding untreated control (respectively 190 fold decrease– 0.024 μm^3^/μm^2^ vs. untreated control 4.56 μm^3^/μm^2^ p =  0.0013; and 2126 fold decrease– 0.005 μm^3^/μm^2^ vs. untreated control of 10.63 μm^3^/μm^2^, p =  0.003). Blank ULV liposomes also resulted into a significant decrease in the biofilm biomass (152 fold decrease; 0.03 μm^3^/μm^2^ for 3 mg/mL ISMN p =  0.0013; and 2650 fold decrease; 0.004 μm^3^/μm^2^ for 60 mg/mL ISMN, p =  0.003 respectively) compared to the untreated control. Comparing ULVs incorporating either 3 or 60 mg/mL ISMN vs. their corresponding blanks however showed no statistical significance using unpaired t-test (p> 0.05). In agreement with our mechanistic study, ISMN alone had a very strong negative effect on the biofilm biomass at a dose of 60 mg/mL (26,575 fold decrease; 0.0004 μm^3^/μm^2^ p =  0.003). There was no significant difference in anti-biofilm effects of ISMN alone compared to ULV at 3 or 60 mg/mL ISMN and their blanks (p> 0.05). These results indicate that both ISMN alone, ULV alone and both compounds combined diminish *S. aureus* biofilms *in vitro* after 24 hours.

### Effects of ISMN-liposome formulations in short term (5 minutes) exposure

**Figure 3:**
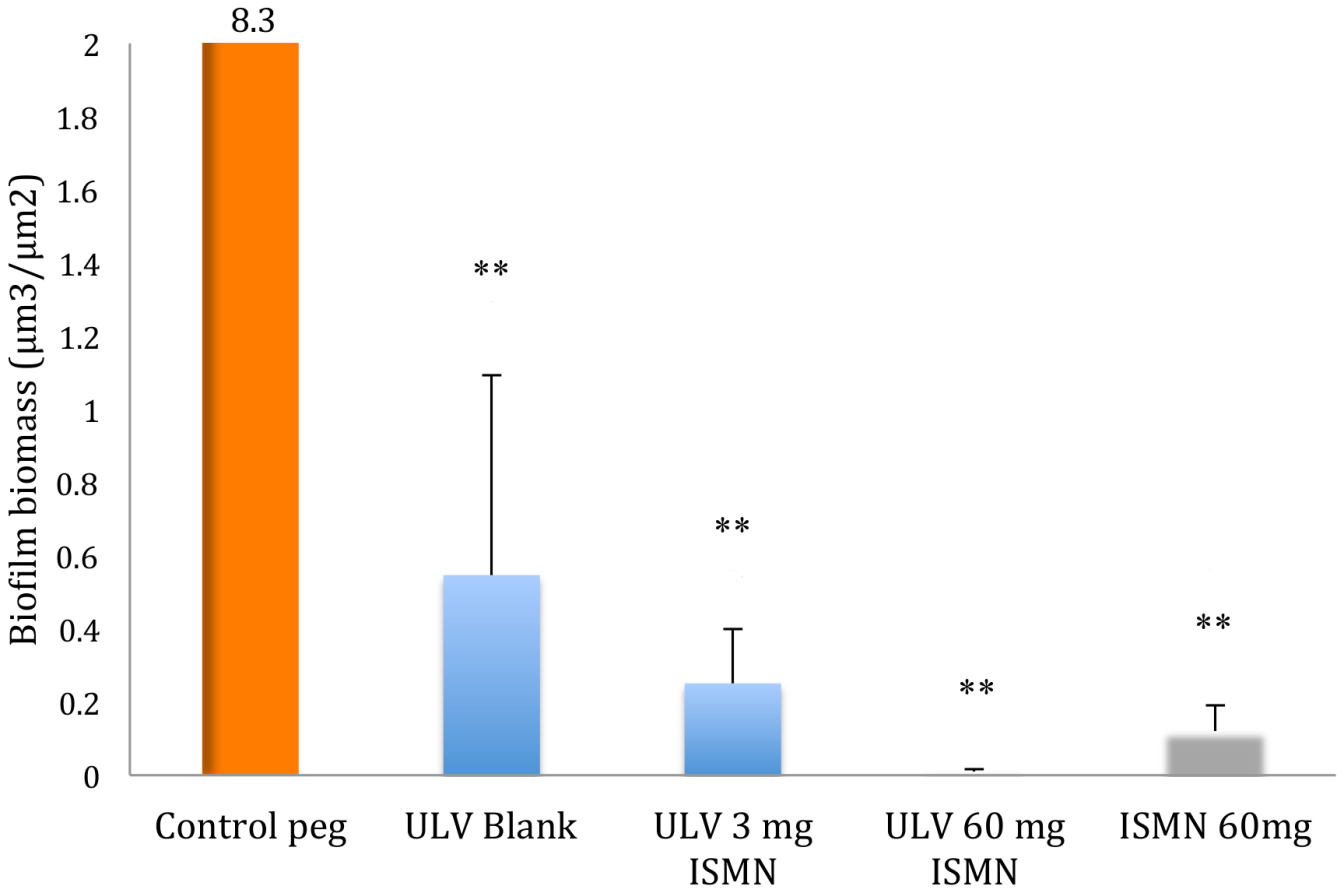
Effects of ULV Blank liposomes, ISMN-ULV liposomes and ISMN on *S. aureus* biofilm biomass after 5 minutes. The number above the bar represents the biofilm biomass beyond the y-axis scale. ULV: Unilamellar liposome; ISMN: Isosorbide mononitrate. Data represents Mean +/– SD of a duplicate experiment. *, P<0.05; **, P<0.01, ***, P<0.001 compared to untreated control; Bonferroni multiple comparisons test.

Based on these results, we wanted to see if the anti-biofilm effects of the above formulations were maintained at a time of 5 min incubation mimicking the shorter contact time of a topical wash into the nose and paranasal sinuses. One-way ANOVA showed a significant decrease in biofilm biomass at 3 mg and 60 mg/mL ISMN ULV liposomes compared to untreated control (respectively 33 fold at 0.25 μm^3^/μm^2^, p =  0.008; and 922 fold at 0.009 μm^3^/μm^2^, p =  0.007 vs. untreated control of 8.3 μm^3^/μm^2^). Blank ULV liposomes also significantly reduced the *S. aureus* biofilm biomass compared to the untreated control (15 fold, 0.55 μm^3^/μm^2^ vs. 8.3 μm^3^/μm^2^, p =  0.009). Although the results did not reach statistical significance, ISMN-liposomal formulations showed stronger anti-biofilm effects than blank liposomes, especially at the higher ISMN dose. The 60 mg/mL liposomal formulation had a greater anti-biofilm effect than the liposome-free ISMN at the same dose (922 fold; 0.009 μm^3^/μm^2^ for 60 mg/ml ISMN ULV vs. 69 fold; 0.12 μm^3^/μm^2^ for 60 mg/ml ISMN alone, p> 0.05). (**Figure 3** **)** Together, these results demonstrate the anti-biofilm effects of ULV liposomes, ISMN free drug and ISMN-ULV liposomes also at short (5 minutes) exposure times, in particular for higher (60 mg/ml) ISMN concentrations.

Building on the above results, 60 mg/mL ISMN liposomal formulations were now selected to investigate the anti biofilm efficacy of this approach at a 5 min incubation duration altering lipid composition. A non-statistically significant decrease was observed in the biofilm biomass when treated with the 60 mg/mL ISMN ULV liposomal formulation at a 5 mM lipid concentration compared to the blank ULV liposome at the same lipid concentration (1.1 μm^3^/μm^2^ vs. 1.7 μm^3^/μm^2^, p> 0.05). However, both groups showed a significantly reduced biomass compared to the untreated control (4.4 μm^3^/μm^2^, p =  0.01 for ULV loaded and p =  0.04 for ULV Blank) but no significant difference compared to the ISMN treated group (1.1 μm^3^/μm^2^). Next a 60 mg/ml ISMN–ULV liposomal formulation at 25 mM lipid concentration was tested. Unlike the lower lipid concentrations, both blank ULV and ISMN ULV liposomal formulations significantly reduced the biofilm biomass (1.1 μm^3^/μm^2^, p =  0.01 for Blank and 0.6 μm^3^/μm^2^ for loaded, p =  0.004) compared to the untreated control. No significant inhibiting effects were however observed compared to the pure-drug ISMN control. Although no statistical difference was attained between the ISMN liposomal formulations at 5 mM and 25 mM, the higher lipid concentration tended to reduce the biofilm biomass to a greater extent than the lower concentration counterpart (0.6 μm^3^/μ 25 mM vs. 1.1 μm^3^/μm^2^ for 5 mM p>0.5).

**Figure 4:**
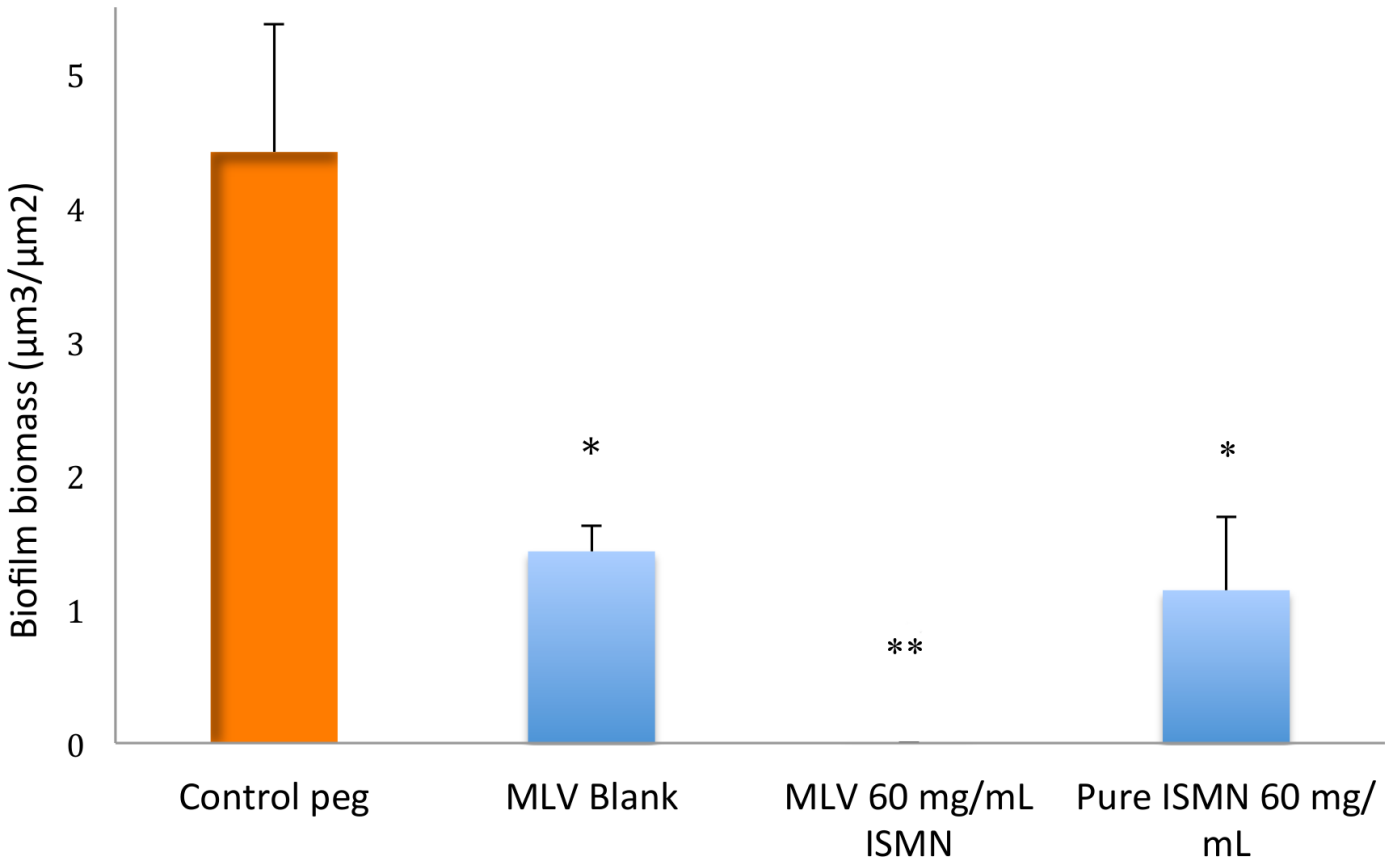
Biofilm biomass of *S. aureus* ATCC 25923 exposed for 5 minutes to MLV liposomes of 25 mM lipid composition. Data shows MLV Blank and MLV liposomes encapsulating 60 mg/mL of the NO donor ISMN. MLV: Multilamellar liposome, ISMN: Isosorbide mononitrate. Data represents Mean +/– SD of a duplicate experiment. *, P<0.05; **, P<0.01, ***, P<0.001; Bonferroni multiple comparisons test.

**Figure 5:**
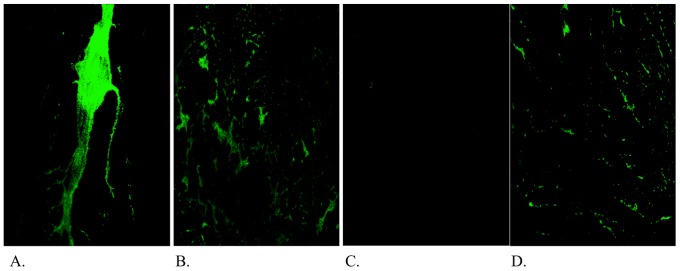
3D projections of *S. aureus* biofilms on pegs exposed for 5 minutes to MLV liposomes with a 25 mM lipid composition. A. Untreated control peg, B. MLV blank liposomes, C. MLV 60/mL ISMN loaded liposomes, and D. 60 mg/mL pure drug ISMN.

Next a 25 mM lipid MLV formulation loaded with 60 mg/mL ISMN was tested and compared to the ULV formulations and untreated and pure-drug ISMN controls. As shown in **Figure 4**, the 60 mg/mL ISMN loaded MLV formulation resulted into a significant decrease in the biofilm biomass in comparison to the untreated control (0.003 μm^3^/μm^2^ vs. 4.4 μm^3^/μm^2^, p =  0.002). Although the MLV formulation had stronger anti-biofilm effects compared to its ULV counterpart at similar ISMN and lipid concentration (0.003 μm^3^/μm^2^ for MLV vs. 0.6 μm^3^/μm^2^ for ULV) the results did not reach statistical significance. This was the same when comparing the MLV formulation with the pure-drug ISMN (0.003 μm^3^/μm^2^ vs. 1.14 μm^3^/μm^2^). The blank MLV also resulted in a biofilm biomass decrease when compared to the untreated control (1.4 μm^3^/μm^2^ vs. 4.4 μm^3^/μm^2^, p =  0.02). Although there was a decrease in biofilm biomass with the encapsulated 60 mg/mL ISMN MLV formulation compared to its blank (0.002 μm^3^/μm^2^ vs. 1.4 μm^3^/μm^2^), the results did not reach statistical significance with the Bonferroni test. **Figure 5** shows representative 3D projection images of *S. aureus* biofilms exposed to the MLV liposomes.

## Discussion

In this study, the anti-biofilm effect of liposomal-formulations of ISMN against *S. aureus* was demonstrated. *In vitro* experiments indeed showed that short exposure times mimicking nasal flush induced a strong reduction in the biofilm biomass. These also showed that modification of liposome size and lipid content dramatically alters its efficacy. Our results may guide which liposomal characteristics are critical in the formulation of liposomes encapsulating compounds for optimal topical delivery to *S. aureus* biofilms.

There is a vast existence of *S. aureus*-related infections where effective anti-biofilm therapy is needed. Infections of the skin,[1] bone,[3] heart (endocarditis),[34] sinuses[7], [11] and device-related infections (catheters, implantable prosthetics)[35], [36] could be targeted with specifically designed liposomes encapsulating the appropriate drug dose. Liposomes, with their phospholipid bilayer, can be modified to deliver drugs to specific physiologic targets to obtain the desired therapeutic efficiency.[37] Alteration of the lipid composition and concentration in the bilayer has been demonstrated in past studies to have significant effects on the extent of adsorption into *S. aureus* biofilms.[38] These liposomal properties thus change the effectiveness of the encapsulated drugs in performing their action. Given the degree of difficulty of biofilm matrix penetration by topical antimicrobials, which originates in a combination of physical and metabolic barriers[39], ensuring that highly efficient drug doses diffuse into and reach embedded bacterial cells, is key for effective biofilm eradication. Bacterial properties such as cell wall hydrophobicity have also been shown to affect liposome penetration and hence drug mobility through the biofilm matrix.[40] In this study, the *S. aureus* biofilm-liposome interaction is yet to be explored and further characterization of this relationship is required.

We chose to use anionic liposomes by virtue of their well-documented anti-biofilm properties against *S. aureus*,[41], [42] with an aim to obtain a synergistic effect with the NO donor ISMN and improve overall efficacy. Furthermore, a comparison of ULV and MLV formulations was carried out. ULV liposomes were demonstrated to have anti-biofilm effects even without the encapsulated NO donor and these effects were more pronounced compared to their MLV counterparts. Although beyond the scope of this study, many factors may alter this antimicrobial effect of bare liposomes. A direct interaction of the liposome with the bacterial cell wall may play a role in better biofilm penetration.[43] Bacterial cell wall properties, different for gram positive and negative bacteria, have been shown to be an important factor in nanoparticle penetration of biofilm matrix.[44] Changing expression of cell-wall proteins can completely switch bacterial surface properties from hydrophilic to hydrophobic without altering biofilm structure, thus significantly altering susceptibility of bacteria to nanoparticles[45] such as liposomes. Thus, characterization of the physicochemical properties of the targeted bacteria is needed to develop specifically designed liposomes in order to produce desired anti-biofilm results. Although the liposomal-bacterial interaction was not characterized in detail in this in-vitro study, a clearer description of this interaction is therefore warranted prior to in-vivo and clinical application.

When comparing the ISMN liposomal formulations of 5 mM and 25 mM lipids, liposomes of higher lipid concentration tended to be superior in reducing the biofilm biomass in comparison to the lower concentration counterpart. In addition, according to the encapsulation study, the drug encapsulation efficiency in liposomes of high lipid concentration was at least 5-fold higher than that of the lower concentration counterpart. It is likely that the encapsulation efficiency plays a significant role in defining the extent of drug delivery to the biofilm. In this study, it was deliberately chosen not to purify the ISMN liposomal formulations towards facilitating their translational uses against CRS. However, this approach further increased the complexity of the system from a structure-activity point of view since both free ISMN and encapsulated drug is present in the formulation. Further mechanistic understanding of how the liposomal formulations affect the delivery of the NO donor to the biofilm is required, as well as elucidating the how the synergistic role of liposomes and ISMN in reducing the biofilm biomass.

Although ULV liposomes have been to date the preferred clinical option considering their optimal pharmacokinetics in blood, in the context of topical treatment such as in CRS, MLV liposomes present many advantages over their ULV counterparts. They are indeed easier to manufacture, and are more stable with longer storage in liquid form. MLV liposomes will thus most likely offer a more straightforward translational path to the creation of a topical sinus rinse. In this study, it was demonstrated that MLV liposomes with greater lipid composition are comparable to ULV liposomes in terms of anti-*S. aureus* biofilm effects encapsulating the same ISMN concentration of 60 mg/mL. Thus in future *in vivo* studies, MLV liposomes will most likely be the approach in the conversion to clinical application in the context of CRS.

Providing alternative antimicrobial agents that are safe and effective, will hopefully contribute to decreasing antibiotic use, and consequently reduce the risk of developing drug resistance. With MRSA emerging as a global concern and a shift in pharmaceutical interest away from antibiotic therapy due to lesser profitability,[46] the development of novel antimicrobials is urgently needed. Despite the necessity to further improve the proposed liposomal ISMN formulations and characterize their mechanisms of action, the identification of the efficacy of high-lipid anionic MLV liposomes proves an important first step in the successful topical utilization of liposomal-encapsulated nitric oxide to treat *S. aureus* biofilm infection in CRS.

## Conclusion

Liposomal formulation of ISMN has significant anti-biofilm effects against *S. aureus*, showing greatest efficacy with higher lipid content in both ULV and MLV systems. Future in-vivo studies are required however, to determine their safety prior to attempts at a topical clinical application.
